# TwitterSensing: An Event-Based Approach for Wireless Sensor Networks Optimization Exploiting Social Media in Smart City Applications

**DOI:** 10.3390/s18041080

**Published:** 2018-04-03

**Authors:** Daniel G. Costa, Cristian Duran-Faundez, Daniel C. Andrade, João B. Rocha-Junior, João Paulo Just Peixoto

**Affiliations:** 1 Department of Technology, State University of Feira de Santana, Feira de Santana 44036-900, Brazil; dca650@gmail.com; 2 Department of Electrical and Electronic Engineering, University of the Bio-Bio, Collao, Region del Bio Bio, Concepcion 1202, Chile; crduran@ubiobio.cl; 3 Department of Exact Sciences, State University of Feira de Santana, Feira de Santana 44036-900, Brazil; joao@uefs.br (J.B.R.-J.); just1982@gmail.com (J.P.J.P.)

**Keywords:** Smart Cities, wireless sensor networks, social media, data mining, event-based optimization

## Abstract

Modern cities are subject to periodic or unexpected critical events, which may bring economic losses or even put people in danger. When some monitoring systems based on wireless sensor networks are deployed, sensing and transmission configurations of sensor nodes may be adjusted exploiting the relevance of the considered events, but efficient detection and classification of events of interest may be hard to achieve. In Smart City environments, several people spontaneously post information in social media about some event that is being observed and such information may be mined and processed for detection and classification of critical events. This article proposes an integrated approach to detect and classify events of interest posted in social media, notably in *Twitter*, and the assignment of sensing priorities to source nodes. By doing so, wireless sensor networks deployed in Smart City scenarios can be optimized for higher efficiency when monitoring areas under the influence of the detected events.

## 1. Introduction

Smart cities have become a central element when addressing several problems in urban environments, such as mobility, public security, energy efficiency and emergency response [1,2]. Actually, the growth of population living in urban environments has brought many challenges for the governments, demanding new policies and strategies to improve the urban quality of life. Smart cities initiatives have then been developed to cope with the increasing concern about efficient resource management, exploiting evolving communication and sensing technologies [3,4]. Currently, Smart Cities are based on the concept of “system of systems”, with different embedded and intelligent devices cooperating to provide valuable information to support a better life in modern cities, operating over Internet-based or application-specific networks. This complex scenario allows the execution of innovative applications, which will strongly support the transformations of the way of living in cities in this century [5].

A sensor is an electronic device endowed with sensing components to measure simple ambient conditions, related to the environment surrounding the sensor. Scalar information such as temperature, humidity, pressure and luminosity can be sensed and processed to reveal some properties about objects, people or events of interest, which can then be exploited by intelligent systems when taking some actions [6]. Moreover, multimedia data can also be sensed when low-power cameras and microphones are embedded in sensor units. The interconnection of sensors in the form of Wireless Sensor Networks (WSN) allows the creation of distributed infrastructures for monitoring in Smart Cities and retrieving information about the behavior of people, the occurrence of events of interest and general conditions about the environment for an uncountable number of applications.

In a Smart City scenario, sensors may be neatly placed to cover well-defined areas, such as roads, public squares and metro stations. In such cases, although energy consumption may not be a concern, since sensors may be supplied by the city’s electrical system, the multi-system dynamic nature of Smart City applications may bring specific challenges that have demanded innovative solutions. Among those challenges, hundreds or thousands of sensors may be continuously transmitting information, which may require considerable bandwidth, especially when streaming multimedia content. Furthermore, real-time delivery may also be required for fast response to external systems requests, which may also demand different levels of reliability guarantees for transmitted data [7,8]. Thus, optimizing transmissions of sensed data in Smart City scenarios is of paramount importance and it is the core motivation of this work.

Modern cities will experience plenty of events during a day, continuously and in an unexpected way, which may have different relevance on the people’s routine and their safety. We can expect that such events may be central in Smart Cities systems. In a superficial analysis, most cities will be concerned with at least traffic conditions, accidents and public security related events, but many more events may also be relevant (e.g., protests, festivals, rainfall level). In such way, wireless sensor networks can exploit the event-centric nature of modern cities to optimize their operation and achieve higher performance. Actually, one way to perform optimizations is assigning priorities to sensors nodes, assuring better resources to more important information [9]. Nevertheless, the identification and classification of events of interests is not straightforward and different approaches may be adopted. In the context of wireless sensor networks, efficient event detection in multi-systems Smart Cities is still a challenging task that has demanded innovative solutions.

Social networks sites, or simply Social Networks or Social Media, have become very popular in recent years due to the increasing proliferation and affordability of Internet enabled devices [10]. This can be concluded looking at the variety of social networks currently available (e.g., *Twitter*, *Facebook*, *Instagram*, *LinkedIn* and many others). Roughly speaking, social media can be defined as a network of interactions of relationships, where the nodes are actors or entities and the edges represent the relationship among them [10]. These interactions contain textual information about everyday events, such as wedding information, sports news or descriptions of a car accident. They may also have spatial information, due to the GPS (Global Positioning System) unit built in recent mobile devices [11]. Finally, temporal information is also present, since the time in which the information is sent is also stored. Analyzing the data contained in interactions, events of interest may be detected, classified and associated to a specific region, providing textual (what happened), temporal (when it happened) and spatial (where it happened) information. According to [12], when these data are efficiently indexed, they can be retrieved in a short amount of time and used in location-based applications.

When people spontaneously post information in social networks about a specific event (e.g., car accident, fire, and robbery), that event could be detected, classified (in a numerical scale of relevance indexes) and associated to sensors inside the region of influence of the event. Sensors in that area may then optimize their operation, for example tagging transmitted packets or changing sensing and coding behavior [13,14]. The efficient detection and classification of events, as well as the proper assignment of sensing priorities to sensor nodes, may open new possibilities for Smart Cities applications, and this promising scenario fostered the development of this work.

This article then proposes a new approach, TwitterSensing, as an external system to be employed in Smart City scenarios, providing a new mechanism to establish sensing priorities to sensor nodes in one or more wireless sensor networks. In short, the contributions of this work are threefold. First, *Twitter* is exploited to provide information about the current events that are happening in any considered city, which is cheaper and simpler than other mechanisms since it only relies on the natural behavior of an arbitrary group of inhabitants in modern cities. Second, a computational approach is proposed and implemented to detect events of interest and to classify them in a numerical scale of relevance. Third, an algorithm and a specialized protocol are designed to establish the computed priorities to the corresponding sensors in the region of influence of the detected events.

The proposed approach will extract information from data flows provided by the *Twitter* database, processing them to detect and classify events of interest, as well as to locate them on predefined urban areas (influence regions of the events). The established priorities may then be exploited for any kind of optimization, but the definition of the optimization approaches is out of the scope of this work.

Actually, this innovative approach may considerably optimize WSN for higher performance and reduce costs, since event detection will be performed exploiting information already present on Internet and spontaneously produced by inhabitants of the city. To the best of our knowledge, such approach has not been proposed before.

**Figure 1:**
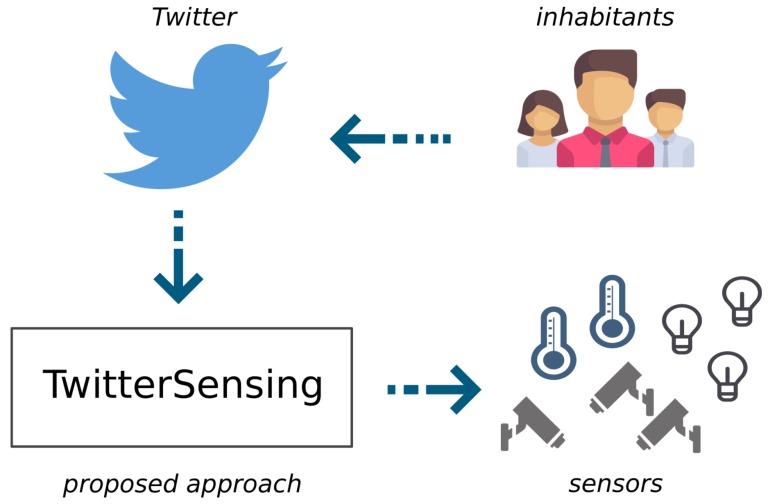
Abstract view of the proposed approach.

The conceptual data flow of the overall solution is depicted in Figure 1.

The remainder of this paper is organized as follows. Section 2 presents the state of the art in areas related to this work. Fundamental definitions are described in Section 3. Section 4 presents the proposed approach. Numerical results are described in Section 5, followed by conclusions and references.

## 2. Related Works

The concept of *Smart City* was created in the context of an increasing demand for resource management in modern cities. In parallel, the constant monitoring of places and objects by interconnected devices originated the Internet of Things (IoT) concept, with objects sharing information autonomously (machine-to-machine communication) [15]. Actually, Smart Cities and IoT have different origins, but they are converging to a common goal of “smart living” [4].

A Smart City is typically comprised of one or more heterogeneous wireless sensor networks, composed of different types of sensors (scalar, video, image and audio). When combined, those sensors provide information about the status of a place or resource. In other words, wireless sensor networks are a relevant component of the backbone of Smart City systems [16].

Independent of the amount of data flowing on the network, WSN should maintain minimum levels of Quality of Service (QoS) to support the city systems. One way to achieve that is giving priority indexes to sensors (sensing relevance) and changing them according to some external or internal parameter (e.g., sensors with better coverage have higher priority). Such relevances can be exploited for a series of network optimizations, providing services as error recovery, congestion control, routing, multipath selection, among others [9]. For example, the work in [17] proposed a generic multiple sinks positioning algorithm based on the sensing relevances of sensors. The relevance level of each sensor node (priority) is considered to calculate the positions of every sink in WVSNs. In a different perspective, the work in [8] exploits the nature of transmitted data to differentiate how corrupted packets will be recovered. Whatever is the employed optimization approach, prioritization levels may be exploited to enhance the performance of the network, delivering high quality data while reducing resource expenditure.

In general terms, we can expect that there are at least two priority-based optimization approaches in evidence on academic and industrial scenario: based on sensor’s properties and based on events of interest. In the first approach, the relevance of each sensor is assigned according to its current properties (e.g., remaining power, location, sensing coverage, and communication characteristics) [9,13,18]. In the second approach, an event (e.g., explosion) can trigger a critical level of monitoring over a region [14,19]. In both cases, events or sensor properties are processed and delivered by the network. However, depending on the number of idle sensors in the network and their processing load, event-based strategies can jeopardize the performance or compromise the network precision while the new priorities are computed and delivered. Despite this costly task, optimizations based on sensing relevance are an effective way to optimize the network without compromising the overall monitoring quality [20]. The presented issues and the trade-off between energy consumption, data quality and network availability may foster the development of new approaches in this research field.

Priorities definition in Smart Cities may then be centered on the detection of events, which is consistent with the particularities of such environment. Actually, automatic event detection has been pointed as one of the main target uses for wireless sensor networks. As sensor nodes normally comprise of computation capabilities, rudimentary event detection is possible by applying some predefined thresholding over the input sensor signal (which includes “on-off”approaches) [21,22], or more developed (still low-complex) local signal processing or decision taking algorithm [23,24]. Note that the last case includes multimedia sensor networks [25], which work with more significant amounts of data, such as in images and sound (see, for example, works in [26,27,28,29]). Moreover, the large-scale distributed nature of WSN has fostered many proposals which consider the cooperation of multiple nodes to detect and follow particular event sources [30]. Indeed, when many sensors are deployed in near places, it is possible that a single event is detected by more than one node. Correctly exploited, this feature can be useful in many ways: first, it helps to prevent possible mistakes in single-node based detection because of measuring imprecisions or unavailability issues; second, it helps to enhance the estimation of the event source location; and, third, it helps to follow the dynamics of the event (movement, extension variations, etc.). Many works allowing event detection and location through WSN have been reported, considering multiple real-world issues, like resource constraints, measurement errors, and information unavailability (see, for example, works in [31,32,33]). Of course, those works can be implemented to successfully allow event detection, with some restrictions in the WSN implementation, like (in most of cases) a dense network topology, enough sensing range or other infrastructure features. In fact, in the existence of WSN infrastructures in Smart Cities, our work attempts to consider an additional layer of information provided by citizens, further improving event detection or even opening new possibilities in this area. Indeed, the many events reported by people (in multiple ways) using mobile devices can be automatically detected and classified for multiple uses, and this concept has been proposed as a potential tool for Smart Cities [34].

Social media have become popular in recent years [10]. Among them, *Twitter* has received a lot of attention on the data mining field due to its real-time nature. In *Twitter*, users share an enormous amount of real-time data containing information about different types of everyday events (e.g., movie premiere, traffic jams, quality of a restaurant) in different contexts and viewpoints. Using data mining techniques, this information can be extracted and used to improve or create new products or services. For example, the work in [35] builds social network models to design more effective marketing plans. However, the real-time nature of *Twitter* also poses challenges in the process of extracting information, such as the dynamic and increasing content and the high amount of trivial information flooding the data flow [36].

Some recent works have exploited *Twitter* to provide relevant information. In [37], using the real-time (textual, spatial and temporal) information spontaneously provided by *Twitter* users, earthquakes and their routes (including the epicenter) can be identified. In that real-life application, the system detected almost every earthquake identified by the traditional ways by just monitoring *tweets* (*Twitter* messages). In another work, tools were provided to analyze the status of public health (e.g., flu or insomnia statistics) and track the evolution illnesses over time [38]. In fact, some events are seasonal or linked to seasonal conditions (e.g., seasonal migration, and increasing of flu rates on cold months) and it follows a pattern (e.g., if flu rate increases in cold months, it decreases in hot months). Using *Twitter*, information were collected and the regularity of several events in a city was measured [39]. Comparing the new events with the reference events, possible unexpected events could be detected in that work.

Other important topic surrounding the analysis of social network data is the targeted advertising. A user of a social network, while using it, submits lots of information to it such as age, visited places, favorite artists and preferred product brands. Usually a text is associated with the mentioned information, providing some value judgment or expectation upon a product, event or service (e.g., a post saying “*My brother will graduate next week, can’t wait for it!*” followed by the user location). The identification of this expectation provides the power to direct a certain product or service linked to user intentions (e.g., if a user is going to a graduation event, he/she maybe would like to know the best hairdresser and clothes store near him/her) [40]. In another perspective, the user data can be used to predict which items would be bought from a given advertiser. Based on the client estimated purchase price for each advertiser, more effective ads are displayed to the user, increasing the profit [41].

Considering the feasibility of data mining in modern social media applications, and the nature of their users, it is possible to extract relevant data about events of interest, which can be mapped to priority indexes to sensors nodes in Smart Cities. To the best of our knowledge, such approach has not been proposed before.

## 3. Fundamental Concepts

Some basic definitions are required when developing the proposed approach, as stated in this section.

### 3.1. Sensing Prioritization

Prioritization is an effective mechanism to improve the overall performance of wireless sensor networks. This because the information transmitted by sensors may have different importance for the overall monitoring quality along the time, and this principle may be exploited. Thus, depending on the nature of the applications, sensors may have different importance levels, e.g., some may be sensing more critical data or they may be responsible for triggering some action. By doing so, the network may provide differentiated services for the most relevant source nodes and their transmitted packets; for example, a transport protocol could prioritize packets from more relevant nodes, assuring faster and more reliable delivery, or a routing protocol could relay more relevant packets through better paths.

Actually, prioritization in wireless sensor networks may have local or global scope and some works have been concerned on optimizations based on one or both of these priority scopes [42]. This work is concerned with global prioritization, since assigned priorities are significant for the entire Smart City scenario. In this work, we define an Event of Interest *e* in a set of events *E* as any event that is relevant to the point of changing the priority of at least one sensor *s* in a set of sensors *S*, increasing or decreasing its priority level. Any event of interest in the proposed approach is detected and classified (associated to a priority level) exclusively processing *tweets*.

The work in [9] proposed a framework that defines priorities as a value within a numeric scale, with 16 different “relevance levels”. In that work, priorities range from irrelevant (0) to maximum relevance (15). We borrow this principle defining that every sensors s∈S is associated to a priority level s.p, a positive integer value between $P_{min}$ (lowest priority) and $P_{max}$ (highest priority), with $P_{min}<P_{max}$. When the proposed approach is initiated, all sensors *s* have the same priority, $s.p=P_{min}$, and the priority of each sensor remains the same until it is explicitly changed due to the occurrence of some new detected event or when it is not anymore under the influence of at least one event (the sensor’s priority is reseted).

### 3.2. Smart City Scenario

Many systems may be deployed in Smart City scenarios, with different particularities. However, for the proposed approach, we are particularly concerned with the perception of a Smart City as any generic system composed of one or more sensors. Actually, any kind of sensors may be considered (e.g., temperature, pollution, UV radiation, audio, image), since the only required information for the priority assignment system is the location of the sensors (managed by the WSN [43,44]). In such way, a Smart City may be represented as a 2D map where a set of *S* sensors are previously positioned, and two sensors are never positioned in the same location.

Therefore, a Smart City SC, sizing $SC_{m}\times SC_{n}$ meters, and assuming (0,0) as the origins at the upper left corner of the rectangle created by SC, is defined as the considered scenario to be optimized. In that scenario, *S* sensors will be deployed, belonging to one or more independent WSN. Each sensor s∈S is located at (s.x, s.y) position, assuming that the dimensions of the sensors are too tiny to be considered when compared to the size of the city, and that $0\leq s.x\leq SC_{m}$ and $0\leq s.y\leq SC_{n}$. It is also assumed that all sensors are fixed and never move, and that they have sufficient processing power to execute the protocols and procedures defined in the proposed approach. The monitoring nature of the sensors are irrelevant in this work, but such information could be considered in future optimizations, employing some strategies based on the type of data gathered by sensors [42].

Finally, it is assumed that all sensors are connected and that they can be reached from outside the sensor network. As sensors may get offline due to energy depletion (of theirs or others required relaying nodes), the list of sensors of the city SC may be updated along the time, but how they communicate and what routing protocols are employed are out of the scope of this work. Actually, as the final goal of the proposed approach is to assign priorities, which are in the last instance assigned on the application logical layer, the employed MAC and routing protocols to compose the ad hoc backbone of WSN are not necessarily an issue to be concerned.

### 3.3. Data Mining from *Twitter*

*Twitter* is one of the most popular “microblogging” tools [45]. In essence, *Twitter* allows the publication of 280-characters limited messages denominated *tweets* sharing any kind of information such as ideas, opinions, impressions or facts about the users’ everyday life. These *tweets* are instantly shared to the user network of followers and, unless previously deactivated, all *tweets* are publicly available. Being a follower of someone on *Twitter* means that the user receives all the messages published by the followed person in his *timeline* and this relationship of following and being followed does not require a reciprocate [46,47].

The reduced size and real-time nature of *Twitter* messages, associated with the asymmetric social relationship of its users, characterizes this social media as a rich source of fresh information rather than other social networking sites [46,48]. Over the last years, *Twitter* has been explored as a distributed social sensor system, allowing real-time detection and tracking of earthquakes [47], detecting patterns on public health [38] or even predicting the stock market [49].

The core of strategies for mining information from the web involves various methods and techniques that originate from “Information Retrieval”, “Artificial Intelligence” and “Natural Language Processing” areas [48]. This sum of different techniques is explained by the complexity and challenges imposed due to the velocity, variety and high volume of the data [50]. In *Twitter* data streams, the challenges are extended due to the short length of *tweets*, frequent and evolving use of informal, irregular and abbreviated words, improper sentence structures and mixed languages. Such characteristics of *Twitter* data makes the traditional text analysis less suitable for *tweets* [51].

The first step of any data mining pipeline is the data collection and consists in collecting the *tweet* corpus that will be mined [50]. In *Twitter*, this task can be done by using their public Application Programming Interface (API). By default, a *tweet* *w* is composed by several attributes or *features* attached to itself, including the text w.D, the spatial information w.A, which is composed by a pair of geographic coordinates (longitude and latitude, $w.A=(w.A_{lon},w.A_{lgt})$) describing the local from where it was sent, the timestamp w.t in which the *tweet* was sent and its corresponding unique id w.id. Other properties, such as author username, language and a list of used hashtags are also present in the *tweet* object, which is organized in the JSON format [52].

The raw data acquired from *Twitter* are noisy and contains lots of uninteresting, missing or inconsistent information. For example, a *tweet* is not always geotagged and the message may contain typos or abbreviated words. Low-quality data leads to low-quality results, thus the data must be preprocessed in order to improve its quality and, consequently, refining and facilitating the mining process [53]. In general, the data preprocessing phase in *Twitter* data mining applications contains the following steps:

- **Feature Extraction:** The raw data extracted from *Twitter* contains features which may not be important to a given mining application (e.g., user profile image url). This cycle consists in choosing which features objects are relevant and will compose the vector of features $w.f=[f_{1},f_{2},f_{3},\ldots ,f_{n}]$ [54]. For example, in most spatial *Twitter* applications [55,56,57,58], w.f=[w.id,w.D,w.t,w.A]. The other features are stored and, if necessary, they can be retrieved in any step by using w.id as identifier.
- **Data Cleaning:** Real-world data tend to be incomplete, noisy and inconsistent. To solve this issue, some entries need to be dropped or some of its entries must be estimated in order to remove major inconsistencies [53,54].

When dealing with *Twitter* data with the previously mentioned features, two major dissonances can be identified. The first one refers to the lack of localization in a large subset of the considered input data. To handle this issue, *tweets* without GPS tags are removed [56,59] or have their location estimated using the user profile information, *tweet* history and entities mentioned in the *tweet* message [60]. The second issue is related to the frequent inclusion of large amounts of informal and irregular sentences and grammatical errors presented in w.D. This unstructured text may leads to poor text analysis that affects the accuracy of analytical algorithms [61]. However, there are some techniques to handle this problem [50,54,62].

The final step of the mining process is to design effective analytical methods from the processed data [10]. Classification and prediction are two forms of data analysis that can be used to extract models describing important data classes or predict future trends [53]. In classification problems, an unlabeled entry *w* is classified into a category by using classification rules extracted from a previously analyzed dataset of objects with the same nature as *w*. For example, a *tweet* can be classified as *positive* or *negative*, indicating if it has positive or negative emotions [63]. Prediction problems, on the other side, aim to perform a numerical prediction related to some event (e.g., estimate how much a client will spent in an online store).

In *Twitter*, studies including both prediction and classification problems were performed. In general, both approaches are combined to achieve a major goal such as real-time event detection [55,56,59] or opinion mining [62,63]. Independent of the problem nature (prediction or classification), methods for analytical processing inevitably involve machine or statistical learning concepts [45,47,63].

Moreover, learning tasks are typically divided into *supervised* and *unsupervised* learning approaches. While *supervised* learning requires tries to predicts the results within a continuous output, *unsupervised* learning produces an output by clustering the data based on relationships among its variables [47]. Usually, supervised approaches are applied for detecting specified behaviors [35,39], while unsupervised approaches are employed in unspecified behaviors [56,59]. However, hybrid approaches can be employed for detecting on-line events which responds to a pre-defined criteria [55].

For the proposed approach, the input *Twitter* data flow is preprocessed before being considered for event detection and classification. In an initial step, the text, geotags, timestamp and id information of each tweet *w* is maintained while the other features are removed, resulting in the vector of features w.f=[w.id,w.D,w.t,w.A] previously mentioned. Before entering the event detector, we extract entities and noun phrases of each w.D using a NLP tool specific for tweets [64] and use them as keywords on the event detection process, which is an analytical unsupervised process discussed on Section 4.2.1. On the other hand, we employ the Multinomial Naive Bayes (NB) supervised analytical procedure on the event grader to classify w.D and obtain its scope value. To tune NB and obtain better results, we apply three common NLP strategies [48,50]: tokenization, stemming and stopword removal. The procedures for scope calculation are detailed in Section 4.2.2.

## 4. Proposed Approach

Considering the high complexity of Smart City systems and the event-based nature of many WSN monitoring scenarios, this article proposes an innovative mechanism to automatically detect, classify and assign sensing priorities to source nodes according to information posted on predefined social media (*Twitter*). This new approach, TwitterSensing, is described in this section.

**Figure 2:**
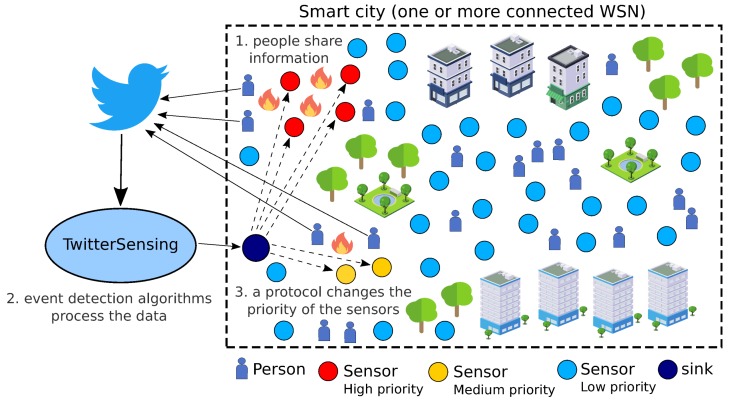
Abstract view of the proposed approach.

Typically, sensor nodes will be deployed to retrieve scalar or multimedia information from the monitored field. Actually, the information gathered and processed by sensor nodes are transmitted toward one or more sink nodes [4,20], depending on the network topology, which may be connected to the Internet. In such way, it is natural to expect that information extracted from the Internet can also be inserted into wireless sensor networks, allowing different kinds of optimizations. This realistic scenario is the basis for the proposed approach and it exemplified in Figure 2.

**Figure 3:**
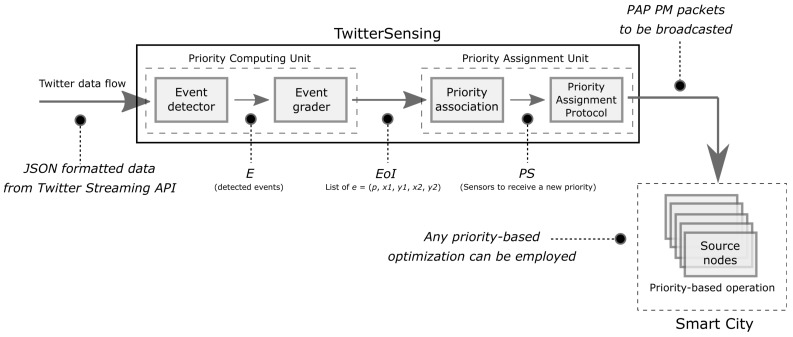
Schematic operation of the proposed approach.

The main goal of the proposed approach is to assign relevance levels (in the form of priorities indexes) to (source) sensor nodes, according to the detection of events of interest from the social media *Twitter*. To accomplish that goal, the overall solution is divided into two logical blocks: (a) Priority Computing Unit (PCU); and (b) Priority Assignment Unit (PAU), which may be executed centrally or not. Both logical blocks of the proposed approach have well-defined inputs and outputs, leading to the final goal of the solution. In such way, considering that TwitterSensing is a logical box that can be employed in different Smart City scenarios and that it is expected to be executed constantly, the input of the system as a whole is a *Twitter* data flow formatted in the JSON standard [52], while the output of the system are messages containing priority information to be inserted (broadcasted) into one or more WSN. Figure 3 presents the overall operation of TwitterSensing.

The PCU block accesses a data flow from *Twitter*, detecting events of interest and classifying them according to their significance for the considered sensing application. For that, the TwitterSensing approach is designed to be located outside the target WSN, as an external system, but it must to be connected both to that wireless sensor network (through its gateway/sink) and to the Internet. The same principle is valid if more than one WSN is considered at once, for a defined Smart City scenario and the same *Twitter* data flow. After computing priority indexes, the PAU block associates sensor nodes to priority indexes and delivers a specialized message to the considered WSN, which should broadcast it to the sensor nodes using any protocol. As the TwitterSensing approach is focused on computing priority indexes to be adopted by source nodes, the way WSN will broadcast priority configuration messages is out of the scope of this article, making the overall solution easier to be adopted by any wireless sensor network technologies.

### 4.1. Events and Sensing Priorities

This work is centered on the definitions of sensors, s∈S, and events of interest, e∈E. For the proposed approach, some additional information is required for sensors and events. Every considered sensor is defined as s=(p,c,x,y); a sensing priority s.p is a positive integer value within a pre-defined range, with $P_{min}\leq s.p\leq P_{max}$ and $P_{min}<P_{max}$, while the value of s.c is used as a persistence variable (further described in this section) and s.x and s.y are the 2D coordinates of the sensor (treated as a point) within a Smart City scenario. On the other hand, an event is defined as $e=(p,x_{1},y_{1},x_{2},y_{2})$; besides the computed priority e.p, an event also defines an area of influence represented by a rectangle with ($x_{1}$, $y_{1}$) and ($x_{2}$, $y_{2}$) as the left-bottom corner and top-right corner coordinates, respectively. The area of influence of any event is of paramount importance, since sensors in that area will be associated to the computed priority.

Sensing priorities are computed based on two characteristics: (a) the potential impact that a detected event has to harm people, defined as *Severity* (sev(e)); and (b) the potential number of people to be affected by the event, defined as *Scope* (sco(e)). Obviously, critical events (e.g., an explosion) in crowded areas are more relevant to be monitored and thus they receive higher priority, when compared with events with low impact on people integrity (e.g., traffic jams). Therefore, a priority e.p is computed considering the *Severity* and *Scope* of the event, as defined in Equation (1). The variable α is used to calibrate the impact of sev(e) and sco(e) on the computed value of e.p. When both parameters have the same impact, α=0.5.

(1) e.p=α×sev(e)+(1−α)×sco(e)

The output of the Priority Computing Unit block is EoI, a list of all detected events *e*, according to the sample frequency employed in the Event Detector Grader module, defined as $\Delta_{t}$. Such a list, defined as ListEoI, may change every $\Delta_{t}$ seconds, according to the processed *Twitter* data flow.

After assigning priorities to source nodes, applications need to define some use for the priority indexes, usually in the form of one or more optimizations [42]. As any type of priority-based optimization may be employed, the TwitterSensing approach does not specify any mandatory optimization mechanism. Actually, every sensor node must implement the adopted priority-based optimization so it can be used. For the sake of numerical verifications of the proposed approach, some reference optimizations will be defined further in this article.

### 4.2. Priority Computing Unit

The PCU logical block is the core element of the proposed approach, since it is responsible for the detection and classification of all considered EoI. Actually, the PCU unit will dynamically access a predefined *Twitter* data stream looking for Events of Interest, which will then be classified and associated to priority indexes. Every detected event will be associated to a single numerical priority value within a predefined range of priority indexes.

The operation of the PCU unit is complex and thus it is divided into two distinct modules: (1) Event Detector; and (2) Event Grader. Both modules are required and they must operate in sequence, as described in the following.

- **Event Detector**: This module receives a real-time data flow from *Twitter* and extracts information from events that are currently happening. This article focuses on *Twitter*, due to its aforementioned real-time nature and high volume of data, but other social media could be considered in future works.
  *Input*: JSON formatted data from *Twitter* Streaming API.
  *Output*: List of detected events (*E*).
- **Event Grader**: After detection, the extracted events data go to this module for classification, where the information is analyzed and, based on a set of predefined parameters (e.g., events related to natural disasters may be more important than party-related events, depending on the applications monitoring requirements), it receives a priority level within a predefined range.
  *Input*: List of detected events (*E*).
  *Output*: List of events associated to priorities (EoI).

#### 4.2.1. Event Detector Module

Event detection from *Twitter* streaming data has been an important topic in academic research, resulting in different techniques with different purposes [47,65]. Based on several contributions in the literature, the most appropriate approach for this work was selected.

Event detectors can be divided into two major groups, as previously described: *unspecified* and *specified* event detection [47]. Referring to these groups, a technique is defined as *unspecified* if no prior information about the event is available. Unspecified techniques monitor the streaming data, detecting bursts on a set of keywords and grouping documents (posts and *tweets*) with similar patterns. On the other hand, a *specified* event detector technique is defined if a query is built from some known event information (e.g., time, location, and description). Generally, specified event detection is applied for retrospective event detection (e.g., [35,38,39]) and monitoring scheduled events (e.g., [66]). In a different way, unspecified event detection is applied for unplanned (also referred to as real-time or on-line) event detection (e.g., [37,56,59]).

Generally speaking, both approaches have advantages and drawbacks. However, whatever are the characteristics of the employed approach, methods for event detection inevitably involve machine or statistical learning concepts [47]. Moreover, learning tasks are also divided into *supervised* and *unsupervised* learning approaches. While *supervised* learning requires tries to predicts the results within a continuous output, *unsupervised* learning produces an output by clustering the data based on relationships among its variables (words, in our case) [47]. In this context, supervised approaches are usually applied for specified event detection [35,39], while unsupervised approaches are employed in unspecified event detection [56,59]. However, hybrid approaches can be employed for detecting on-line events which responds to a pre-defined criteria [55].

A Smart City can be assumed as a universe of uncertain parallel events. In such way, due to the amount of variables involved on those events, the monitoring costs involved and the unspecified nature of the events, an unsupervised event detector is the most appropriate choice to the problem of this work. Moreover, the real-time nature of the proposed adaptive sensing relevance approach requires a real-time event detector and, in order to properly reach the sensors and deliver the priority configuration, the event detector should be capable to indicate the region in which the event is happening (e.g., a minimum bounding rectangle involving the affected region). This information is essential to associate sensors to the computed priority.

In Table 1, unspecified real-time event detection approaches are compared, focusing on the previously discussed requirements: “Proposal” refers to the application main goal, while “Structural Content” details the provided information about a detected event.

The applications in Table 1 are divided into two major groups: *global* and *local* event detection approaches. Global event detectors aims to recognize unusual behaviors from the entire *tweet* stream (e.g., world premiere of a movie), while local event detectors intent to recognize events happening in small geographic regions (e.g., a city festival). Thus, a global detection method applied over a stream of geotagged *tweets* tends to miss many local events [56]. As mentioned above, this article requires an high precision event detector, capable to detect events originated in small areas (e.g., city park or any neighborhood). Consequently, global event detectors do not meet our requirements.

Analyzing the presented approaches in Table 1, there is some information that is relevant to this work. First, despite Jasmine’s good ability to detect events, its main goal is to allocate geotags for non-geotagged *tweets* [60]. Based on the *tweets* location (latitude and longitude), Jasmine extracts the entity which represents where the event is happening (e.g., a concert at *House of Blues*). By restricting the event geographical information to one representative place, Jasmine does not consider the region impacted by the event. In EvenTweet [59] and GeoBurst [56], the location is represented by the spatial distribution of *tweets* related to events.

The EvenTweet strategy to detect events includes a continuous analysis of the most recent *tweets* within a time-based sliding window [59], enabling the tracking of events over time. These time windows have a fixed length and the event detection is triggered only when the current window is saturated, compromising the real-time event detection [56]. Moreover, to detect a keyword burst, EvenTweet clusters the keywords only according to their spatial distribution, resulting in irrelevant keywords in the cluster. Due to this strategy, that system is not capable to detect two distinct events happening simultaneously in the same location [56].

Finally, in a different way from EvenTweet, GeoBurst uses spatio-textual clusters to group incoming *tweets*, organizing them into geographically close and semantically coherent clusters. This strategy generates candidate events which are further ranked according to spatio-temporal burstiness, eliminating fake events (e.g., routine activities) [56]. In addition, GeoBurst has an update module which enables real-time and continuous event detection when the time window shifts [56]. Considering its effectiveness in front of other applications, this work exploits the same strategy of GeoBurst [56] to implement the Event Detector module.

In a general form, GeoBurst is an effective method for performing a real-time local event detection in localized tweet streams. A local event is an unusual activity triggered in a delimited area and restricted number of participants in a specific location (e.g., protests, fires, and soccer matches) [56,59]. An event naturally boosts the production of geo-tagged tweets around the event epicenter (e.g., fans at the stadium posting tweets about the soccer match), these tweets are geographically close (near to the stadium) and semantically coherent (they all give an information about the soccer match), forming a “geo-topic cluster” and characterizing a potential event [56]. A geo-topic cluster is not necessarily a local event because it may represent a routine activity (e.g., art-related tweets sent from a museum) or an occurrence of a global event (e.g., the premiere of a TV show may spread lots of geo-topic clusters throughout the city). Thus, in order to identify the local events and rank them, the spatiotemporal burstiness of each geo-topic cluster is measured [56].

The first step of GeoBurst is to gather all geo-topic clusters as candidate events in a given query window. To achieve this, the “geo-topic authority” of each tweet is obtained by combining the geographical and semantic contributions from similar tweets. In short, the geographical contribution is measured by using the Epanechnikov kernel function, while the semantic contribution is calculated using random walk on a keyword co-ocurrence graph [56]. Afterwards, an authority ascent process is executed in order to find all high authority tweets (pivots). Pivot tweets attract similar tweets to form geo-topic clusters [56]. Finally, the candidates are ranked by its spatiotemporal burstiness. To achieve this, the data stream is continuously summarized in a efficient structure denominated “activity timeline”. The summaries presented on this timeline describes the routine in different regions, serving as background knowledge to measure the spatiotemporal burstiness and select the true local events [56].

#### 4.2.2. Event Grader Module

A city scenario is susceptible to a wide range of events (e.g., crimes, floods, traffic jams, protests, accidents, and explosions) and each one has a different nature and impacts the citizens routine in different ways. People posting direct or indirect information about such events on *Twitter* may contribute to optimizations on any sensor-based monitoring systems, once events are properly detected and classified.

Actually, each event that may happen has a risk (or priority) and this risk level is defined by two factors: the event scope and the event severity [69]. Based on Bostrom’s scope/severity grid, five contextual severity levels are established and a method to calculate the scope of events was created, as presented in Table 2.

The “Contextual severity” directly refers to the event nature and is obtained by analyzing the event keywords, while the scope refers to the sensitivity of a spatial region and is obtained by analyzing the average population (number of *Twitter* users) in the area reached by the event.

Analyzing Table 2, one can see that “Unbounded” is the event type with the highest severity level. Actually, city public celebrations and big private events (e.g., New Year at Times Square and Billy Joel show at Madison Square Garden) impact many people (e.g., fans, merchants, neighbors, and police) and causes the reconfiguration of its neighborhood for a certain period of time (e.g., traffic redirection). This event type must be carefully watched, because floods, fires, explosions, car crashes, among other disaster-related events can quickly impact many people in this region, requiring immediate actions to relieve the impact and prevent consequences, for example providing health care, redirecting the traffic and call the fire department in the case of a fire. For the second group, “Social” related events refer to small and non-periodic events such as company parties and ceremonies. This events are restricted to a small region, but it may indicates a forthcoming unbounded event (e.g., *event described by a message saying “Pre-concert dinner and drinks with my friend!”*). “Weather” events usually involve large regions but they consequences are less harmful than previous events due to the lower density of people in the location. “Mobility” events are characterized by movement of considerable groups of people, which describes the city routines and average behavior (e.g., series of check-ins on Central Park on a Saturday). Such events are mostly harmless, but they require attention due their capability to describe the usual flow of people across the city. Finally, “Imperceptible” events cause no damage nor changes in the city routine (e.g., a global event missed by the event detector) and are discarded in the classification process.

To perform the extraction of the contextual severity (sev(e)) from a event *e*, we ran the event detector over a section $W_{t}$ of our complete dataset *W*, obtaining $E_{t}$ events. Each $e\in E_{t}$ were manually tagged by observing the text of its corresponding pivot tweet $w_{p}$. The manual classification process was performed three times, in different days, and the final tag of each event was defined as the most common classification for that event and, in case of an inconsistency on the tags, a definitive classification is performed. Afterwards, the textual information of each $w_{p}\in E_{t}$ is properly preprocessed to remove noisy information by using common NLP techniques: a twitter-aware tokenizer [70]; Snowball Stemmer to remove morphological affixes; and a process of stopword removal [70]. Further, each processed tweet is converted into vector representation $w_{p}.f$ using term frequency-inverse document frequency (TF-IDF) [71] and fed, as training data, into a Multinomial Naive Bayes classification model which is used to determinate the contextual severity of a event. In general, the Naive Bayes (NB) algorithm has a high recall and precision while classifying textual data, being one of the most suitable algorithm this task [50,62,72].

The same event may have different associated risks according to its scope. In other words, a city is composed by several facilities and each one has its own routine. For example, a given bakery is probably crowded in the morning, while most nightclubs tends to be empty at daytime and crowded at night. Thus, an event happening at rush hour of a place (e.g., crime near a nightclub at night) is more important than an event happening while the place is under its capacity. To increase the priority precision, providing different approaches to events with the same nature in different regions, the “scope” is defined as the average number of *Twitter* users on a certain area at the event moment, giving a good perception of the expected impact on people.

To estimate the average population of a given area in a specific time window, it is assumed that the considered city (associated with the processed *Twitter* data flow) follows a weekly routine (e.g., beaches are most frequented on Saturdays and Sundays). Following this premise, the city historical data is grouped according to the weekday in which the *tweet* was sent, resulting in seven different data clusters. Further, a spatio-temporal query [57] is performed on the corresponding cluster (e.g., cluster for Saturday *tweets*), returning the number of users in the given region in different weeks. The average value of the result set is defined as the event scope value, which is an important information to compute the priority of the events.

For a time window $\Delta_{t}$, a set *E* of events is detected and fed into the Priority Computing Algorithm (PCA), defined in Algorithm 1. The PSA is responsible to calculate, scale and combine the priority components. Thus, for each event e∈E, the contextual severity (sev(e)) and the associated scope value (sco(e)) are measured by using the previously mentioned techniques and attached to the event descriptor (lines 3–4). After that, the algorithm performs a normalization on the scope value by a factor of the maximum sev(e) divided by the maximum sco(e) (line 6), resulting in similar numeric scales for both severity and scope values. On line 8, we combine the grades obtained by the event weighted by a parameter 0<α<1, resulting in the event priority (e.p). On line 9, the event descriptor *e* is stored in a max-heap EoI . After calculating the priority of all events, EoI is returned containing all detected and classified events.


**Algorithm 1:**
*Priority Computing Algorithm (PCA)*


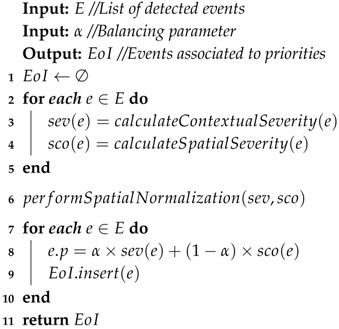



### 4.3. Priority Assignment Unit

After detecting events of interest, with their respective priorities and areas of influence, the Priority Assignment Unit takes place. For that, the list EoI is transmitted from the PCU to the PAU every $\Delta_{t}$ seconds to allow the association of sensor nodes to the corresponding priorities.

The operation of the PAU unit is complex and thus it is divided into two distinct modules: Priority Association and Priority Assignment Protocol. Both modules are required and they must operate in sequence, as described as follows:

- **Priority Association**: This module processes a list of detected and classified events of interest and associates the priority of those events to a subset of sensors.
  *Input*: List of events associated to priorities (EoI).
  *Output*: List of sensors to be assigned to priorities (PS).
- **Priority Assignment Protocol**: This protocol is proposed to define how priorities will be transmitted to sensors. Initially, this protocol specifies a single message type to be broadcasted over one or more WSN.
  *Input*: List of sensors to be assigned to priorities (PS).
  *Output*: PAP PM messages.

#### 4.3.1. Priority Association Module

Sensor nodes are scattered over a Smart City to retrieve some kind of information. As defined before, it is assumed in this article that sensor nodes are fixed and do not move during the network operation time. For any event $e=(p,x_{1},y_{1},x_{2},y_{2})$, a sensor s=(p,c,x,y) may be located inside or outside the rectangle defining the influence area of an event *e*. If the sensor s∈S is inside that rectangle, it receives the same priority associated to that event (s.p=e.p).

The Priority Association module computes a list of all sensors with the corresponding priorities, PS. To reduce the number of information that has to be inserted into the network, only modifications of priorities need to be transmitted. For that, PS contains only the sensors that need to have a new priority assignment, since sensors in the network keep the same priority until a new priority assignment is performed (priorities do not expire).

When a list of events is received from the Priority Computing Unit every $\Delta_{t}$ seconds, defined as EoI, the Priority Association module processes it for all sensors, associating each of the selected sensors to the proper priority, as specified in the Priority Association Algorithm (Algorithm 2). One should note that the PAA is valid for a single WSN, but, for multiple wireless sensor networks, this algorithm has just to be executed for each considered network, since the proper parameters are supplied.


**Algorithm 2:**
*Priority Association Algorithm (PAA)*


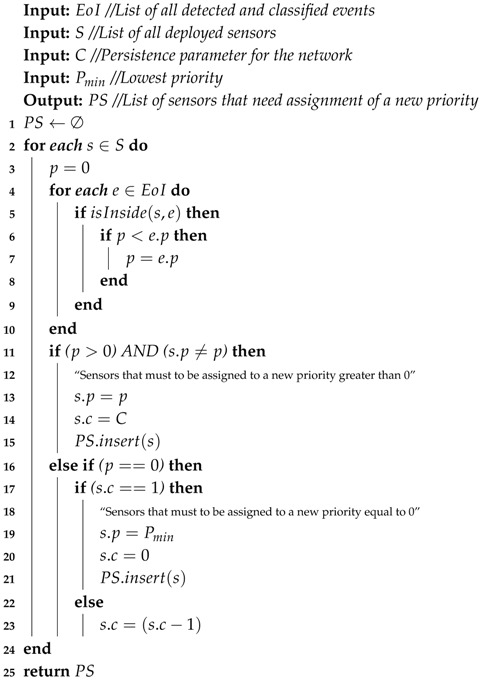



As can be seen in Algorithm 2, sensors are always associated to the highest priority of the detected events (line 13). If a sensor is inside the area of influence of more than one event of interest (line 5), it is associated to the highest priority value among those events (lines 4–10). This is performed because sensors must be associated to the priority of the most relevant event, in the case sensors are under the influence of more than one event of interest, keeping the network operation reasonable for event-based optimizations.

To avoid unnecessary transmission of messages over the network, only new priorities (lower or higher) will be assigned to sensor nodes. In other words, if a sensor is under the influence of only an event in time *t* and it is under the influence of only the same event on time $t+\Delta_{t}$, it is not necessary to “refresh” the assigned priority to that sensor node, since priorities do not expire. By doing so, the number of control messages transmitted to the considered wireless sensor network is reduced. The PAA does not change the values of s.p and s.c of such sensors, nor it includes those sensors in PS.

When an event is detected, sensors inside the rectangle of the area of influence of that event are associated to the event’s priority. It is natural to expect that sensors will keep that priority until they are associated to a new event. However, if a sensor is not associated to at least one event, it should be assigned to the lowest priority, $P_{min}$ (line 19). In such cases, even if a previously detected event is no longer detected, because, e.g., fewer *tweets* are being posted, sensors should “persist” with the last defined priority, once the transmitted contents may be still relevant for the monitoring application. This principle is defined in this work as “sensing persistence” and modeled with the variable s.c. For the Priority Association Algorithm, when a new sensing priority is assigned to a sensor, which can be higher or lower than the previously assigned priority, the value of *c* is set to *C* (line 14). Actually, *C* is a positive integer constant that indicates for how many executions of the PAA the sensor will keep the assigned priority even when it is not associated to at least one event. In other words, it indicates how many $\Delta_{t}$ cycles the sensor will persist with the priority (decremented on line 23), until it is finally assigned to $P_{min}$.

The Priority Association module returns PS, which is the input for the Priority Assignment Protocol. The PS is a subset of *S* with all sensors that must to be assigned to a new priority, lower or greater than the previous priority.

#### 4.3.2. Priority Assignment Protocol

After detecting events and defining their priorities, sensors are associated to priority levels. Every $\Delta_{t}$, a new PS will be created and, if it has at least one element, the real sensors have to be notified about the new priority they must use. Actually, there are different ways to send messages to the proper sensors, with different particularities. Whatever the case, we are particularly interested in mechanisms that consume few resources and that present scalability and low-complexity characteristics.

First, the PAA computes a PS list that contains the information of zero or more sensors, using the formalisms adopted in this article. Although not explicitly defined, every sensor has a generic “id” that is not necessarily understood by the considered wireless sensor network, but that may be easily mapped to a valid id or network address, according to the employed MAC and routing protocols. However, how it will be performed is out of the scope of this work, making the TwitterSensing approach suitable for any communication technologies and network designs.

An important research area of wireless sensor networks is sensors reconfiguration [7,73]. Actually, after nodes deployment and initial operation, sensors may need to be reconfigured to perform some additional or different task. To allow reconfiguration of sensor nodes, special control messages have to be delivered over the network, informing about the requested reconfiguration. We borrow this principle to define the Priority Assignment Protocol (PAP), which is responsible to notify sensor nodes about a new priority value to be used. Although the TwitterSensing approach does not necessarily define a reconfiguration, the same principle may be employed, once a new parameter value will be assigned.

The operation of PAP is based on broadcast of a specific control message. As PS contains only the sensors that need to change their priority value, it may be transmitted on few messages (maybe only one). Message broadcasting in wireless sensor networks is often implemented and already supported by many protocols [74], since basic services such as “querying” are usually supported in many WSN applications. Finally, such solution keeps the proposed approach scalable and independent of the employed MAC and routing protocols in the considered wireless sensor networks.

The Priority Assignment Protocol defines a single message, defined as the Priority Message (PM). It is a simple message with *N* Priority Fields, with each of these fields composed of 4 bytes. The first two bytes is reserved for the “id” of the sensor, resulting in $2^{16}$ possible values. Actually, the “id” field may be mapped to network addresses by the sink, but due to compatibility reasons, the operation details were left outside the definition of the Priority Assignment Protocol. The second field of PM is the “priority” field, which also sizes 2 bytes. Together, the “id” and “priority” fields are able to transmit all necessary information in PS for the considered WSN. Nevertheless, different strategies could also consider the values of s.x and s.y to locate sensors and assign the proper priority, according to the adopted sensors localization strategies [43,44].

Finally, it is worth mentioning that all sensors must to be able to process PAP PM broadcast messages, but only sensors indicated in the PAP PM message will consider the corresponding value in the “priority” field.

## 5. Results

The proposed approach is intended to be used in modern cities as an effective mechanism to support the creation of more efficient wireless sensor networks, which is valuable when multiple concurrent WSN applications will be in execution in urban scenarios. As it works on a complex scenario, which exploits tweets for event detection, the validation of TwitterSensing is not straightforward. Nevertheless, we assessed the performance of this approach in different aspects, as presented in next subsections.

### 5.1. Event Detection and Classification

Initially, it was desired to attest the effectiveness of TwitterSensing concerning detection and classification of events of interest. For that, a considerable large tweets database was used as an input for a series of tests. After that, some reference optimizations were defined, demonstrating the practical usage of sensing prioritization.

The experiments were performed over one dataset obtained using the *Twitter Realtime Filter* [75]. The obtained dataset ($W_{NY}$) consists of 1.74 million geo-tagged tweets sent from New York, one of the most active cities on *Twitter*, from 7 February 2017 to 19 May 2017. Each tweet contains the textual information (message), location (latitude, longitude) from which the message was sent, the time stamp when the message was sent, and identifiers of the author of the tweet. Besides the spatial window, no other constraint was applied when collecting the data.

The Event Detector was executed over the entire $W_{NY}$ considering $\Delta_{t}=2$ h, obtaining |E| = 20,046 events distributed across the city of New York. To perform the event severity classification, we built two disjoint sets $E_{p}$ and $E_{t}$: first, we extracted the first four weeks of *E* to build the training partition $E_{t}$, resulting in $|E_{t}|=8211$; the remaining set $E_{p}$ comprises 10 weeks, from 0 to 9 and starting on 6 March 2017, and composes the evaluation partition which will be considered on the studies of this section. While building our model, we first considered a baseline Multinomial Naive Bayes (NB) classifier with no optimizations nor preprocessing steps over the input data and, gradually, we expanded it by including preprocessing tasks to increase the model hit rate. By the end of this process, after including all previously mentioned NLP techniques, we achieved an average score of 71.02% correctly classified events over 64.69% of the raw NB performance. When compared to other baseline algorithm using Support Vector Machines, another common approach for text classification, the average baseline rate drops to 42.43%. In all mentioned steps, we employed an 10-fold cross validation procedure. After including our best classifier on Algorithm 1, it was calculated the priority of each $e\in E_{t}$. The output of this process is discussed in this subsection.

**Figure 4:**
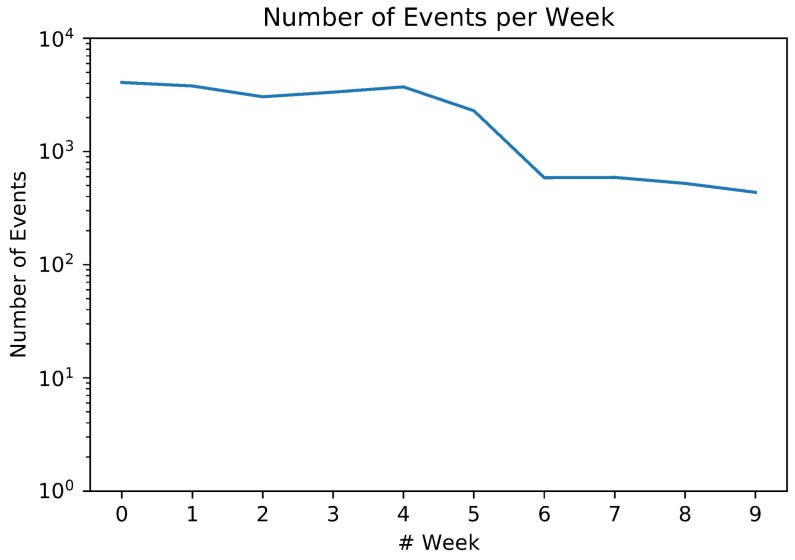
Schematic operation of the proposed approach over the considered tweets dataset.

Figure 4 shows the number of detected events per week in a logarithm scale. Ranging from 4076 detected events on Week 0 to 436 on Week 9, this number is highly correlated with the amount and quality of the input data. Since the detection process is composed by a geographical and semantical analysis, a lower number of detected events is related to the lack of events itself, to a lack of spontaneous report from the users regarding some currently happening events or to highly odd messages about the same event jeopardizing the semantical analysis. In our dataset, we have a notable drop in the rate of detected events starting in Week 5 and stabilizing on the next weeks. The inflexion point, Week 5, correspond to the last week of April where we may have an wide combination of features (e.g., ending of vacations).

**Figure 5:**
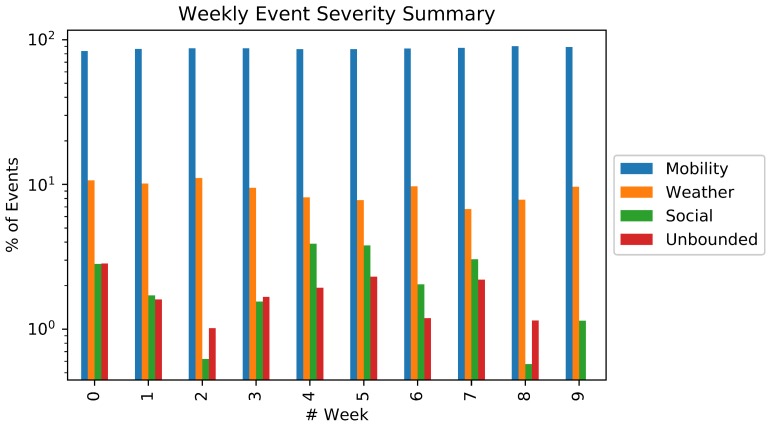
Weekly Event Summary.

Besides the number of detected events, it was also assessed the percentage of tweets according to their classification. Figure 5 shows the percentage, in logarithm scale, of events by week grouped by their severity. Mobility-related events are dominant over all weeks, ranging from 83.63% on Week 0 from 90.44% on Week 8. On the other hand, unbounded events are the least frequent events, having weeks with no occurrences of them (Week 9). Despite some similarities, each week has a distinct event signature characterized by notably small changes in the event severity distribution, which directly reflects the city itinerary during the respective period and is essential for performing WSN optimizations. For example, events such as “shows” and “protests” of Women’s History Month [76] triggered the high level of unbounded events on Week 0.

**Figure 6:**
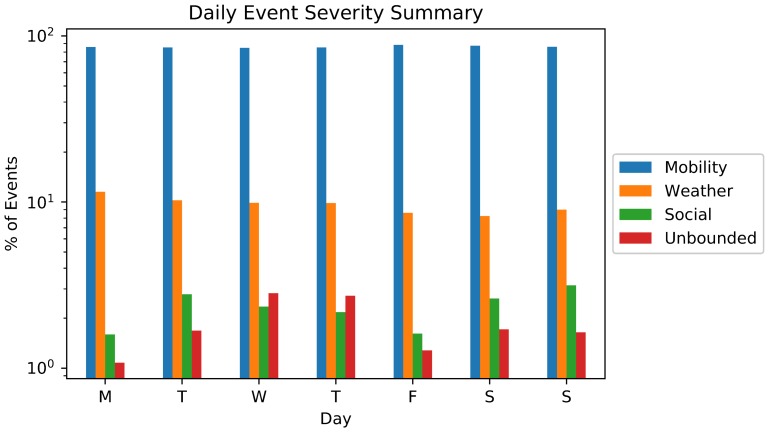
Daily Event Summary.

Figure 6 shows the average event severity distribution by weekday in logarithm scale, clarifying some general properties of Figure 5. Foremost, independent of the weekday, Mobility and Weather related events are the most frequent in any weekday. On the other hand, unbounded events are dominant over Social events on Wednesdays and Thursdays, indicating a clear centralization of impacting events during this workdays which may highly impacts the economy, traffic and behavior of essential services depending of the event location and scope.

The results presented in Figure 5 and Figure 6 depict an interesting behavior of modern cities (at least in New York), indicating the types of events that will happen along the time. Such kind of verification can provide static information that can support better designing of sensor-based monitoring systems.

**Figure 7:**
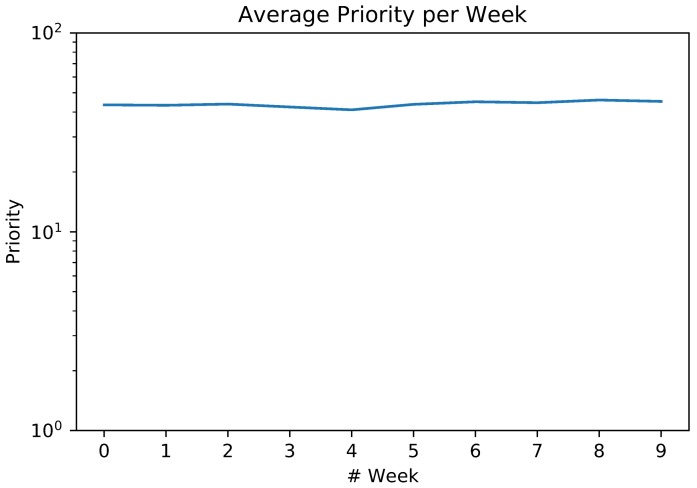
Average Priority per Week.

For the considered dataset, the average event priority by week computed by the Priority Computing Unit was also computed, as depicted in Figure 7, according to the proposed formulations to calculate a priority value for every detected event. In the presented results, the maximum average value is correspondent to Week 8. Compared with Figure 5 and Figure 6 we perceived that, despite the slightly variation on the severity distribution which implicates in a small standard deviation (1.44), the weekly priority has a clear central tendency near to p=43.86 due to the scope influence, which reinforces our initial city weekly routine hypothesis.

**Figure 8:**
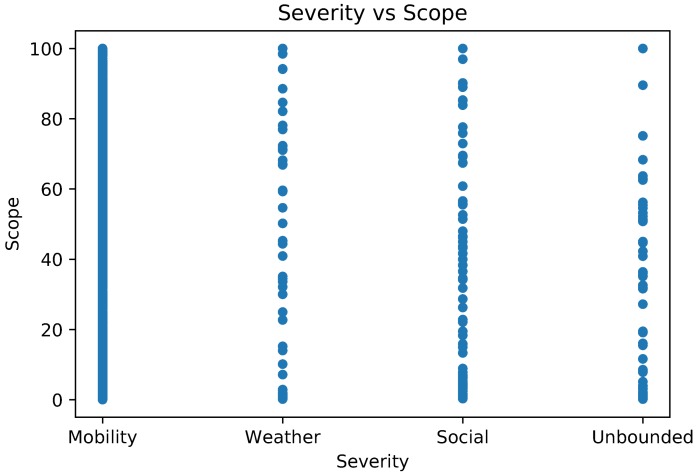
Scope in function of Severity.

This relation of severity and scope in $W_{NY}$ is studied on Figure 8. While mobility events covers almost the entire scope spectra, weather events are concentrated in very small scope values with more than 90% of them having a scope value inferior to 20. Social events are widely distributed, with 37.45% of its events having scope values between 30 and 70. Unbounded events have a similar distribution, with 48.19% of its events concentrated in the same scope interval.

These characteristics show the essential role of the scope, since the events which would be unfairly associated with very similar priorities even with very different risks are now weighted by this factor.

**Figure 9:**
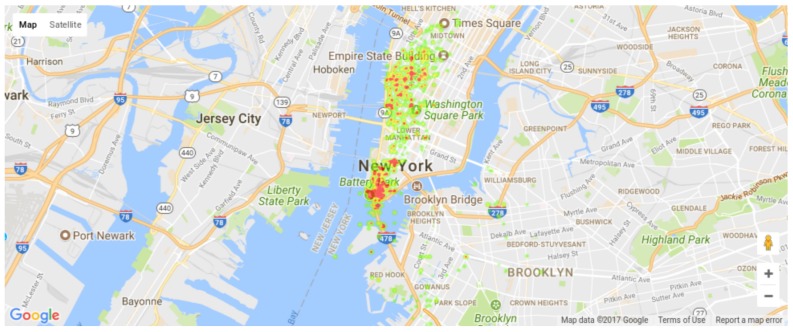
Priority distribution in Weeks 0 and 1.

**Figure 10:**
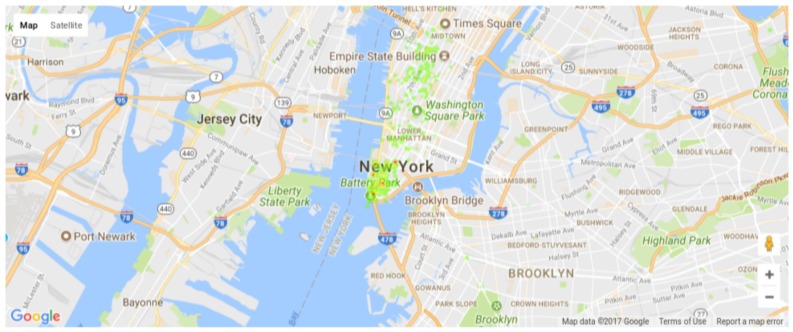
Priority distribution in Weeks 6 and 7.

In a different perspective, the positions of the detected events are checked for the city of New York. Figure 9 and Figure 10 present heat maps of the detected events weighted by the respective priorities in different weeks, plotted using the Google Maps APIs [77]. Figure 9 depicts the events of Weeks 0 and 1, having a large number of unbounded events across Manhattan and few scattered events in Brooklyn, showing that specific regions (e.g., Battery Park neighborhood) may have more monitoring demand than momentarily quiet regions according to the events happening near it. The city of New York has a well-know uneven population distribution, where Brooklyn and Manhattan are the most populated regions. It is interesting to notice that New York’s detected event distributions is slightly similar, where Manhattan and Brooklyn naturally generated more relevant events. In short, the Priority Computing Unit has very interesting results for densely populated regions, being directly linked to the population assiduity in Social Medias.

Actually, the tests showed two different uses of *Twitter*-based detection of events of interest. First, the detection and classification of events of interest is an effective mechanism for priority computing and assignment for sensors in WSN applications, as proposed by the TwitterSensing approach. Second, if large tweets datasets are considered, statistical information can be used to support the decision of where to deploy sensors. By doing so, regions with historical occurrence of more relevant events may indicate that more sensors should be deployed there, or even sensors with more resources (processing capability and energy supply) should be considered for deployment in that region.

After performing the tests, we could see that event detection exploiting geo-tagged tweets is feasible for Smart Cities, mainly when there are many *Twitter* users in the considered region. The detection and classification of events using the proposed approach may then be used to assign priorities to sensor nodes, opening new possibilities of optimizations for WSN applications.

Although the practical use of *Twitter* has been validated to provide information about events in a city, as well as their priority for sensing monitoring applications, the considered dataset was processed offline (batch processing). However, online processing of tweets can be easily performed, which is indeed required for quick and dynamic assignment of priorities to sensor nodes. As the performed tests were concerned with the practical detection and classification of events, large offline datasets were considered, but online processing of tweets is straightforward using the proposed Priority Computing Unit and the public *Twitter* API.

### 5.2. Exploiting Priority for Optimizations

In general, the events that were detected and classified by the Priority Computing Unit will be somehow exploited by one or more wireless sensor network, since the expected outcome is the enhancement of the overall monitoring performance. For that, priority indexes will be associated to sensor nodes (by the Priority Assignment Unit) and broadcasted into Priority Messages. After proper assignment, sensors may operate in different ways, depending on the application monitoring requirements, and thus how they will perform is out of the scope of this work. However, some priority-based optimizations may be defined as a reference, providing some hints about how sensors’ priorities may be exploited.

In general, global QoS parameters as event-based prioritization may be exploited in many ways and during distinct stages of the network lifetime. Different results may be achieved depending on the design of the proposed optimization approach and thus it is not easy to say that some particular optimization will always be better for the network performance. Nevertheless, for this particular verification, a reference approach was defined: SmartCitySensing. This optimization approach is a simple mechanism that exemplifies how sensing priorities may be exploited.

The SmartCitySensing approach was defined to allow that sensors adjust their sensing behavior in a Smart City scenario, according to the assigned priority. As long as applications define a monitoring profile, the sensing behavior of each sensor node may be a function of the computed priority level. Table 3 presents the mapping for the SmartCitySensing approach, associating priorities to transmission patterns.

**Figure 11:**
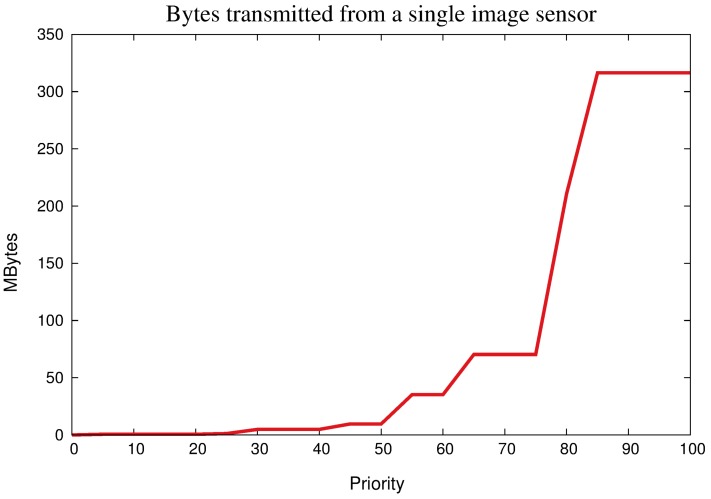
Transmissions from a single image sensor according to the assigned priority.

The definitions in Table 3 are just references, since any configuration may be defined. In fact, the idea is to provide higher monitoring quality for sensors with higher priority, since the increasing in the visual data quality may be usually achieved when more information is transmitted (depending on the application monitoring requirements). Actually, the reduction in the transmission flow for lower relevant sensor nodes may be obvious according to the definitions in Table 3, but we computed the amount of transmitted bytes for a single sensor node along the time, for transmission of only image packets, as expressed in Figure 11. It was assumed that every pixel is represented by 16 bits and that image packets where transmitted for 60 s.

As can be seen in Figure 11, higher priorities will result in the transmission of images with higher resolution and with higher frequency, which may be also thought as an increasing in visual monitoring quality for higher values of priority. In this situation, optimization is achieved when sensors are differentiated according to the detected events, since the expected quality of visual information should be a function of the relevance and area of influence of detected events of interest. In overall, transmission bandwidth usage is reduced and energy is saved since it is not necessary to apply the same transmission pattern (high visual monitoring quality) for all sensors, which could be required for networks where all sensors have the same relevance.

Actually, many different event-based optimizations may be designed and there are some examples in the literature [3,9,42]. Whatever is the chosen optimization, the most critical issue is the proper computation and assignment of sensing priorities to source nodes, and this relevant part can be efficiently performed employing the proposed approach.

### 5.3. Employing TwitterSensing in a WSN

The previous subsection presented a simple but enlightening approach that demonstrates how sensing priorities can be exploited to optimize the network operation. Actually, as sensors will acquire different transmission behaviors due to the established priorities, the network resources are used in a more efficient way, optimizing the network as a whole. The conducted validation is only a first step in this direction, leading us to perform more complex tests to assess the effectiveness of TwitterSensing.

**Figure 12:**
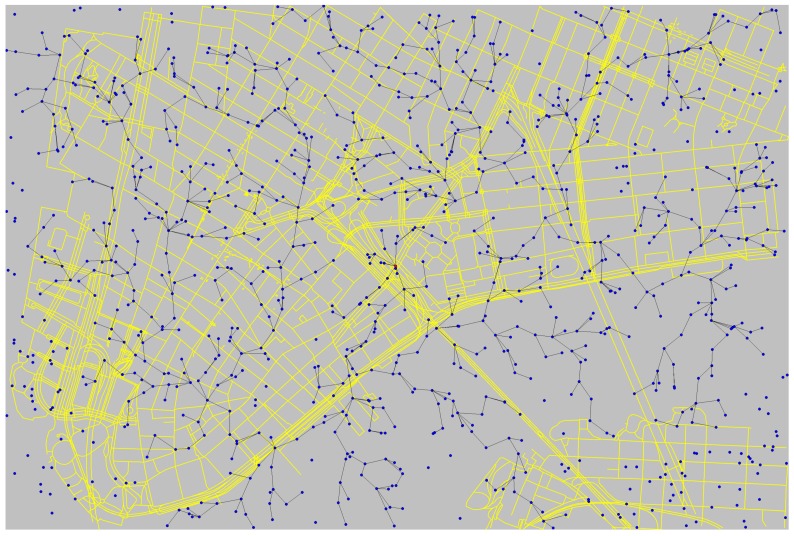
Subregion of Manhattan that was considered for the simulations. In total, 1000 sensors were randomly deployed.

Considering the dataset retrieved from New York City and that was already processed for event detection and classification, the flow of the computed priority values was considered in a simulated wireless sensor network, further supporting validation of the proposed approach. A subregion of Manhattan with area of 6 km^2^ was considered, as presented in Figure 12, where 1000 sensors were randomly deployed. For this simulation, the tool developed in [17] was adapted to process a continuous flow of priority indexes, which is processed to establish priorities.

The simulated sensors are configured to have a communication range of 100 m and thus some sensors are offline after deployment, as can be seen in Figure 12. The communication paths are established based on this communication range and a simple graph-based shortest-path algorithm (lines in Figure 12), and all packets are transmitted toward the sink, which is located at the center of the considered region.

**Figure 13:**
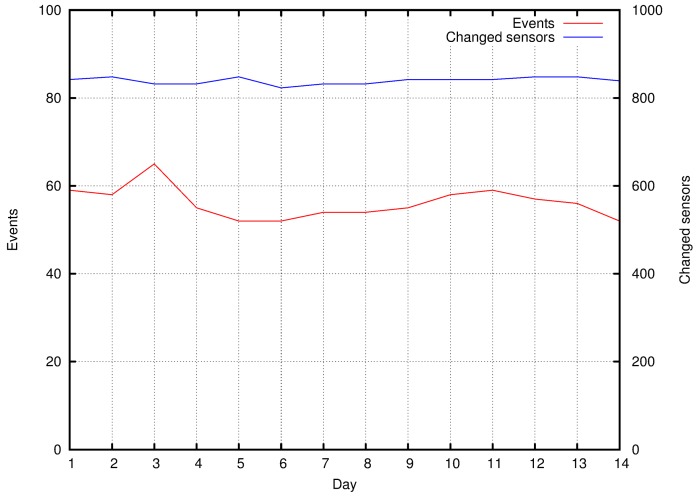
Detected events and number of sensors that will change priority.

Based on the scenario presented in Figure 12, and considering the input data provided by the developed TwitterSensing approach, different results could be achieved. As an initial validation, the impact of detected events on the number of sensors that will receive a new priority index was assessed, as presented in Figure 13. For this verification, 14 days were considered from 7 February 2017, which is a subset of the processed data from New York tweets that was taken in Section 5.1.

As can be seen in Figure 13, on average, more than 50 events were detected and classified every day, and many sensors were affected by those events, which means that they received a new priority index by the proposed TwitterSensing approach. For the considered *Twitter* dataset, there is a daily occurrence of events that could be exploited to adjust the priority of the sensors.

**Figure 14:**
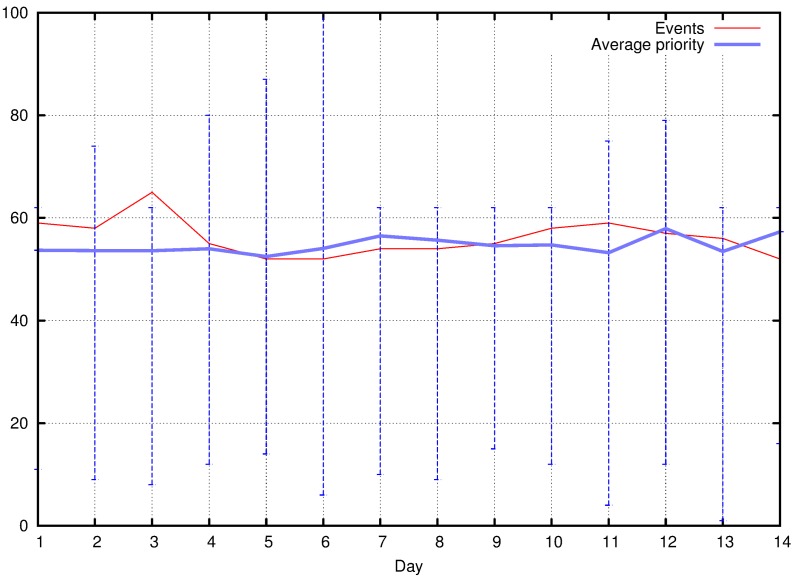
Detected events and average computed priorities.

Besides the number of events, the average computed priority for the same period of time was also assessed, as presented in Figure 14. The computed average priority is also presented with the margin of error, allowing us to better notice how priorities were computed for the considered time. Actually, the average computed priorities for two weeks were almost the same, but there are significant differences when considering the highest computed values (as on Day 6 in Figure 14).

Obviously, the priorities of the computed events depend on the considered day and the configurations of the Priority Computing Unit, but as the priority range for the tests is set from 0 to 100, one can see the dynamical computation pattern for the considered city and period of time.

Actually, the performed verifications gave an important perception of the applicability of the proposed approach in a real scenario, reinforcing the expected benefits when employing the TwitterSensing solution for Smart City optimizations.

## 6. Conclusions

Smart cities are expected to be one of the dominant platforms in the near future, integrating many heterogeneous Internet of Things applications to accomplish different goals in urban scenarios. This complex scenario will be permeated with events that may be relevant to many applications. Actually, exploiting this characteristic may improve the performance of many applications in this area.

The proposed approach uses *Twitter* messages as the basis for event detection and classification, which is indeed a key element of prioritization in wireless sensor networks. As people spontaneously post information that is directly or indirectly related to events, the TwitterSensing approach provides an effective mechanism that can be used in real Smart City scenarios. As an expected outcome, one or more WSN applications can be optimized, contributing to the overall performance of the system.

The proposed approach can have additional applications that were not initially thought. In fact, TwitterSensing proposes a practical way to change network behavior (parameters) to enhance quality of WSN services, based on the location and nature of the reported events. However, by using the proposed system, it is also indirectly possible to detect weakly covered areas where the presence of new sensors is required, which is also an important contribution for many scenarios. Obviously, TwitterSensing can be mixed with other event detection systems to reinforce the estimated nature of the reported events, enhance network quality of the service, and enhance information quality. Such an integration is out of the scope of this work, and can be considered as a future work.

Although the initial results are promising, many more verifications should be performed. Initially, as additional future works, complementary tests over different sets of tweets will be executed. Time issues and real-time operation of the proposed system will be evaluated, considering the total time between event detection and final priority assignment to sensor nodes, which represents the complete operation cycle of the proposed approach. Such test is relevant, but complex, since it is related to the assessment of the real-time behavior of the proposed approach, and thus it was left for future works. Actually, the implementation of TwitterSensing in a real urban environment may bring more complete results, further validating this article.

## Figures and Tables

**Table 1:** Some unspecified *Twitter* on-line event detection applications.

Application	Proposal	Structural Content
EvenTweet [59]	Local event detection	Location; Start time; Keywords
GeoBurst [56]	Local event detection	Representative *tweet*; Related *tweets*
Jasmine [60]	Local event detection	Location, Start time, Keywords
TEDAS [55]	Global event detection	Event score; Location; Start time; Related *tweets*
Twevent [67]	Global event detection	Keywords
TwitterNews [68]	Global Event detection	Representative *tweet*

**Table 2:** Contextual severity levels for prioritization of events.

Contextual Severity (sev(e))	Event Type	Example
100	Unbounded	City Christmas party, big show
75	Social	Movie premiere, company party
50	Weather	Rainfall, snow
25	Mobility	Visit to Central Park
0	Imperceptible	Global events

**Table 3:** SmartCitySensing approach.

Priority	Transmission Pattern
**Image resolution**	
p=0	no image
0 <p≤ 25	176 × 120 (QCIF)
25 <p≤ 50	352 × 240 (CIF)
50 <p≤ 75	640 × 480 (VGA)
75 <p≤ 100	1280 × 720 (HD)
**Image frequency**	
p=0	no image
0 ≤p≤ 20	0.25 image/s
20 <p≤ 40	0.5 image/s
40 <p≤ 60	1 image/s
60 <p≤ 80	2 images/s
80 <p≤ 100	3 images/s
**Video transmission**	
0 ≤p≤ 25	H.264 VGA 15fps
25 <p≤ 50	H.264 HD 15fps
50 <p≤ 75	H.264 VGA 30fps
75 <p≤ 100	H.264 HD 30fps
